# Genetic distance from wolves affects family dogs’ reactions towards howls

**DOI:** 10.1038/s42003-023-04450-9

**Published:** 2023-02-06

**Authors:** Fanni Lehoczki, Attila Andics, Arik Kershenbaum, Enikő Kubinyi, Daniela Passilongo, Holly Root-Gutteridge, Friederike Range, Vicente Palacios Sánchez, Lori Schmidt, Simon W. Townsend, Stuart K. Watson, Tamás Faragó

**Affiliations:** 1 Department of Ethology, Eötvös Loránd University, Budapest, Hungary; 2 MTA-ELTE ‘Lendület’ Neuroethology of Communication Research Group, Budapest, Hungary; 3 ELTE NAP Canine Brain Research Group, Budapest, Hungary; 4 Department of Zoology, University of Cambridge, Cambridge, UK; 5 Girton College, University of Cambridge, Cambridge, UK; 6 MTA-ELTE Lendület “Momentum” Companion Animal Research Group, Budapest, Hungary; 7 Department of Veterinary Medicine, University of Sassari, Sassari, Italy; 8 Animal Behaviour, Cognition and Welfare Group, Department of Life Sciences, University of Lincoln, Lincoln, UK; 9 School of Psychology, University of Sussex, Brighton, UK; 10 Domestication Lab, Konrad Lorenz Institute of Ethology, University of Veterinary Medicine, Vienna, Vienna, Austria; 11 ARCA, People and Nature, SL, Oviedo, Spain; 12 International Wolf Center, Ely, MN USA; 13 Department of Psychology, University of Warwick, Coventry, UK; 14 Department of Comparative Language Science, University of Zurich, Zurich, Switzerland; 15 Center for the Interdisciplinary Study of Language Evolution, University of Zurich, Zurich, Switzerland; 16 Department of Evolutionary Biology and Environmental Studies, University of Zurich, Zurich, Switzerland

**Keywords:** Animal behaviour, Behavioural genetics

## Abstract

Domestication dramatically changes behaviour, including communication, as seen in the case of dogs (*Canis familiaris*) and wolves (*Canis lupus*). We tested the hypothesis that domestication may affect an ancient, shared communication form of canids, the howling which seems to have higher individual variation in dogs: the perception and usage of howls may be affected by the genetic relatedness of the breeds to their last common ancestor with wolves (‘root distance’) and by other individual features like age, sex, and reproductive status. We exposed 68 purebred dogs to wolf howl playbacks and recorded their responses. We identified an interaction between root distance and age on the dogs’ vocal and behavioural responses: older dogs from more ancient breeds responded longer with howls and showed more stress behaviours. Our results suggest that domestication impacts vocal behaviour significantly: disintegrating howling, a central, species-specific communication form of canids and gradually eradicating it from dogs’ repertoire.

## Abstract (web summary)

Domestic dog breeds lose the wolf-like vocal and behavioural responses connected to howling as they become more genetically distant from wolves.

## Introduction

Howling is a long-distance communication signal common in the *Canidae* family^1,2^, though it has been best studied in wolves^3–7^. As a long-distance call, howling has a dual function: (1) the localisation and cohesion of pack members and (2) maintaining the territory and avoiding contact with unknown individuals^3,6,8,9^. For example, howls are used when two parts of the same pack locate each other before a reunion, when individuals at the den (pups, helpers) communicate with other pack members at a distance, before going on a hunt, etc.^10,11^. Howls can be emitted by a single individual, referred to as solo howls, or as a coordinated group vocalisation, referred to as chorus howls^10,11^. Solo howls of wolves are various in length (0.5–11 s) with a fundamental frequency (*f*0) between 150 and 780 Hz^6^, and they often contain frequency modulations. Chorus howls are more complex and often start with an elongated initial solo howling. They take 60 s on average and get more complex with time: on the one hand by the growing number of individuals joining the chorus, on the other hand by the growing *f*0 variability of individual calls in the chorus rendering them acoustically different from solo howls^5^.

Responding to howls is essential for localising other pack members and avoiding physical contact with unfamiliar wolves^3,9^. However, wolves’ tendency to respond can depend on several factors, including season^12^, social status, age, presence of resources like pups or kills^3^, pack size^3,13^ and locality^3^. Studies show that a response can be elicited by playbacks or even human imitations of wolf howls; in these cases, the general response rate varies between 13% and 78%, depending on the factors mentioned above^3,4,12,14^. In addition to producing their own howls, wolves may respond to howls with a variety of behaviours, including attention, approach/avoidance, agonistic/submissive behaviour, greeting, scratching, raised leg urination, lying down, and other vocalisations like barking, whining, growling^13^.

While howling is the hallmark vocalisation of wolves, and several studies address its use and function, about dogs, the closest living relatives of wolves, we know only that they can use various howl types^15^. However, we know nothing about how they use and perceive howling and, consequently, how domestication may have affected this behaviour. The domestication process has altered not only morphological^16^ and physiological features^17,18^, but also, behavioural traits of dogs: e.g., socio-cognitive skills^19^, reproductive behaviour^20^, cooperativeness, aggression^21–23^—which may be linked to each other and is commonly referred to as domestication syndrome^24,25^. Selection for tameness and higher stress tolerance acted on genes involved in the development of the neural crest^17^. For example, genes associated with the human *Williams–Beuren syndrome* (WBS) play an important role in domestication-related morphological (e.g., craniofacial shortening) and behavioural (e.g., hyper-sociability) changes and also influence other genes (e.g., FOXP2), were previously linked to vocal behaviour^26^. This process was caught in the act in the famous experiment done on silver foxes (*Vulpes vulpes*)^27^, where researchers found that domestication syndrome traits appeared as by-products of the artificial selection for high social tolerance and low aggression against humans^28,29^ while the vocal behaviour of these foxes also changed dramatically (the usage rate and context of different call types)^30,31^. Domestication left its mark on dogs’ communication style too, both in their repertoire size and its components^1,32^. Hence their communication differs from that of wolves, reflected for example, in the presence of various types of barking^33^.

However, the more recent selective breeding for different purposes has affected dog breeds in divergent ways, resulting in differences not only in their appearance but also behaviour^34–37^. Breed formation is thought to be a two-step process. In the first step, more than 500 years ago, the focus was on the function of the dogs, with several possible backcrossing events with wolves, forming breeds referred to as ancient (or basal). This category includes 13–16 breeds, like Shar-Pei, Basenji, Akita, or Saluki. All other breeds, so-called modern breeds, were created in the second step approximately during the last 200 years when humans started to breed dogs for different specialised purposes with a standardised appearance^38–40^. Consequently, ancient breeds share more common genetic features with wolves than modern (or recent) breeds do^38–41^. This genetic similarity with wolves is reflected in various behaviours of these breeds^42–44^, and, at the same time, differentiates them from modern breeds^45^. This indicates that the behavioural features preferred by humans, or, in other words, the selection pressures acting on dogs, may have changed during the second step of breed formation, further shaping modern breeds^46^.

The number of studies investigating the possible differences in the vocal communication of ancient and modern breeds are thus far limited. There is one study that directly compared different dog breeds’ vocal behaviour: Feddersen-Petersen reported that some breeds like Alaskan malamutes and Kleiner Münsterländer have very similar vocal repertoires to wolves, while others, like American Staffordshire Terriers and Poodles, have a reduced repertoire size^47^. Beyond this more than 20-year-old publication, only barking but no other vocalisations were explicitly investigated between different breeds: the results of these studies suggest that ancient breeds use it seldom and in limited contexts, while modern breeds use it excessively^33,36^, which is somewhat in line with the result of the study of Feddersen-Petersen.

Although previous studies on separation-related behaviour reported that howling might appear in most breeds’ vocal repertoire^48,49^, and recently suggested to have genetic determination^50^, we predict differences between ancient and modern breeds. In the anthropogenic niche lacking a pack structure, howling has lost its primary functions as a territorial and cohesion signal; thus, modern breeds might not use howling in a functional (if any) way. Nevertheless, ancient breeds may still retain some of the original functionality of howls due to their genetic closeness to wolves and/or admixture with wolves^39^. Group-working sleddogs are especially interesting since a recent genetic analysis suggests that they originated even earlier (>9500 years ago) than previously thought and probably also crossbred with ancient Siberian wolves^51^. As some studies already demonstrated behavioural similarities between ancient breeds and wolves and differences between ancient and modern breeds^42–45^, we can expect a difference also in their reactions towards wolf howls, the ancient breeds being more wolf-like than modern breeds. In line with this, we can expect that dogs’ reactions towards the howling of their closest living relative, the wolf, also correlate with the breeds’ genetic distance from the last common ancestor (root distance). Therefore, we expect that breeds genetically closer to the common ancestor, compared to more distant modern breeds, will react more strongly to howls, reflecting the evolutionary history of breeds.

To test this prediction, we designed a playback experiment presenting dogs with wolf howls in a lab setting. We expected differences along the root distance in vocal responses reflecting mainly the dogs’ emotional state (aggression, fear, stress, etc.), and differences in stress-related behaviours. We assumed that wolf howling might be perceived as an agonistic, territorial-defence signal given that the dogs were in an unfamiliar testing location; hence dogs might consider themselves as intruders in this context. Previous studies showed that wolves use two different strategies in such a situation, which we expect to be similar in dogs: replying with howls, which can help to deter the resident pack, or escaping silently^4^. As our tests were conducted in a confined space without the possibility to escape, we assumed that they likely lead to behavioural signs of stress in dogs, such as yawning, mouth licking, urinating, etc.^52^ and/or contacting the owner looking for comfort^53^. Due to the higher genetic similarity, we expect that dogs with lower root distance will have a better understanding of wolf howls, thus, we expect more howling responses and more stress reactions in more ancient than in more modern breeds.

The howling behaviour of dogs may also vary due to individual features unrelated to their breed’s genetic relatedness with wolves. Indeed age, sex, and reproductive status might have an effect on the vocal behaviour of the dogs. In wolves, it was shown that vocal responsiveness to howls increases with age and is presumably higher in male individuals^3^. Furthermore, studies suggest that the presence of old individuals is important for the success of territory defence^54^, which can be related to the increasing level of agonistic interactions with age (probably due to higher experience and more willingness to engage in conflict as older wolves have more to lose), but this was shown only in males^55^. In dogs, several behaviour traits are affected by individual features like sex, reproductive status and age^56–60^, which might also affect reactions to howling. For example, territorial and dominance-aggression are higher in males than females and in middle-aged dogs than in young or old ones^57,60^. Intact males and neutered females show a higher level of aggression and other behaviour problems, like stimulus reactivity^59^. Females and neutered dogs show higher fear reactions to loud noises than males and intact dogs, and there is also a trend in older dogs compared to adult ones^61^, which is in line with the finding related to personality change, namely that boldness is decreasing with age^56,58^. Based on the results of wolf and dog studies above, we expected that intact animals, male and older dogs respond to howls more likely and show stronger stress indicating behavioural reactions.

The type of howling (single or chorus) may also influence the dogs’ response, as the acoustic structure and the function of these two are different^6,7^, thus we aimed to test their effect on the dogs’ responsiveness. We hypothesised that chorus howls and single howls following the chorus elicit a stronger response than single howls played before the chorus due to sensitisation.

Thus, to test our predictions, we conducted a study with 68 purebred dogs moving freely in the testing room in the presence of their passive owner with a 3-min-long sequence of single and chorus wolf howls presented from a hidden speaker.

We recorded the dogs’ reactions on video, from which we coded behaviours related to attention, stress, owner contact, exploration, and their vocalisations. We analysed the frequency of stress behaviours and calculated principal component (PC) scores from the time percentage of the other behaviours. We ran two separate analyses to test the effect of breed and, thus, genetic relatedness with the last common ancestor with wolves. First, we applied the more traditional ancient vs modern breed categorisation based on the clads provided by Parker et al.^38^, to ran an analysis to test the breed effect more conservatively. Furthermore, to get a finer view of how domestication and later breed selection affected the dogs’ behaviour, and to control for sample imbalance due to the higher number of modern breeds, the root distance of a breed (calculated based on the consensus tree provided by Parker et al.^38^) was used as a continuous scale variable during the analysis. However, we refer to breeds with shorter distances as ‘more ancient’ and those with longer distances as ‘more modern’ breeds for readability’s sake. We used dogs’ age as a continuous (scaled) variable in the analysis, while sex and reproductive status were two-level grouping factors.

We predicted that dogs of more ancient breeds would have a more wolf-like response pattern, showing a higher tendency to vocalise (howl, moan or whine, specifically) and more stress-related behaviours to wolf howl playbacks relative to modern breeds. Conversely, we predicted that more modern breeds would show fewer stress behaviours or reply behaviours due to the lower genetic relatedness with wolves, leading to a different perception of howling. Besides the breeds’ genetic distance from wolves, we predicted that sex, reproductive status, and age influence the dogs’ responsiveness. Specifically, as the responsiveness and aggression of wolves are higher in males, increase with age, and in dogs, males—especially the intact ones—show a higher level of dominance and aggression while invading a territory, we expect a higher level of behavioural responses in intact males, and an increase with age.

Our results indicate that dogs’ behavioural response to playbacks of wolf howls is predicted by the individual’s age and genetic distance to their last common ancestor with wolves. Older dogs of more ancient breeds respond with howls longer and show more stress-related behaviours too, while more modern breeds seem to react rather with barking. These findings support the hypothesis that domestication changed dogs’ vocal repertoire, and through the course of selective breeding by humans, dogs’ responses to howls changed fundamentally. These results shed new light on how selection might affect the vocal behaviour of species on both the perception and production sides. It was known already that artificial selection during domestication could change the structure of vocalisations^62–64^, but our findings are among the first ones indicating that domestication can alter how animals process and react to others’ vocalisations. This, ultimately, might help us better understand the evolution of vocal communication.

## Results

### PCA scales of the time percentages of the behaviour

Based on the principal component analysis, we formed seven scales capturing the general behaviours of the dogs during the playback. These were labelled as: (1) Attention, (2) Reply vocalizations (3) Standing or moving, (4) Avoiding the exit, (5) Contact with the owner, and (6) Agonistic vocalisations. For the detailed composition of the scales, see Table 1.

**Table 1:** Distribution of the coded behaviour variables between the PCs.

	Attention	Reply	Standing or moving	Avoiding the exit	Contact with the owner	Agonistic vocalisations
Listening	**0.816**	−0.190	−0.023	0.009	0.058	0.001
Orient to the sound	**0.814**	−0.052	0.083	0.269	−0.149	−0.030
Head tilt	**0.618**	−0.023	0.286	0.126	−0.117	−0.105
Explore lab	**−0.544**	−0.122	0.276	**0.415**	−0.213	−0.151
Bark-howl	−0.112	**0.799**	0.053	0.105	0.029	0.105
Howl	−0.071	**0.686**	0.069	0.048	−0.061	**0.416**
Yelp	0.001	**0.668**	0.057	−0.069	0.200	−0.288
Moan	−0.079	**0.665**	0.011	0.006	−0.209	−0.192
Lie	0.002	−0.062	**−0.854**	0.016	−0.126	−0.029
Stand	0.293	0.259	**0.737**	−0.210	0.027	0.037
Move	**−0.437**	−0.221	**0.563**	0.332	0.007	−0.076
Proximity to the owner	0.145	0.234	**−0.468**	0.308	0.234	−0.048
Orient to the exit	−0.255	−0.026	−0.011	**−0.814**	−0.134	−0.070
Proximity to the exit	0.017	−0.142	0.358	**−0.681**	0.021	−0.035
Touch owner	0.005	0.072	0.027	0.075	**0.798**	−0.140
Orient to the owner	−0.127	−0.133	0.108	0.033	**0.774**	0.109
Growl and Growl-howl	−0.075	0.019	0.099	0.032	0.007	**0.765**
Woof	0.055	−0.037	−0.055	0.039	−0.040	**0.726**
Proportion Variance	0.134	0.126	0.119	0.092	0.083	0.083
Cumulative Variance	0.134	0.261	0.379	0.472	0.555	0.638

The most important items (>0.4, the item inclusion threshold) within a component are highlighted in bold.

### Vocal reactions

#### Descriptive analysis of the vocal reactions

Of the tested dogs, 57.3% (39 of 68 individuals) reacted with vocalisations in response to wolf howls (for definitions of call types, see Supplementary Data 4). These vocal reactions were classified and labelled as: (1) Reply vocalisations, defined as the sum of howls, bark-howls, yelps and moans, (2) Agonistic vocalisations, which consist of growls, growl-howls and woofs, based on the PCA results, while (3) Stress vocalisations including whine and whine-howl, and (4) Owner directed vocalisations, which mean barks based on their communicative function^33,49,65^. Table 2 summarises the frequency of different vocal responses.

**Table 2:** Frequency of different vocal reactions.

Vocal category	Vocalisation types	Occurrence in the tested dogs	
		*N* dogs	% dogs
Reply vocalisations	Howl, Bark-howl, Moan, Yelp	18	26.5
Agonistic vocalisations	Growl, Growl-howl, Woof	9	13.2
Stress vocalisations	Whine, Whine-howl	32	47.1
Owner directed vocalisations	Bark	18	26.5
Dogs showed vocal reactions		39	57.4
All dogs		68	

The following results contain all dogs, regardless of whether they vocalised or not during the behaviour tests.

#### Reply vocalisations scale including howl, bark-howl, moan, and yelp

**Fig. 1:**
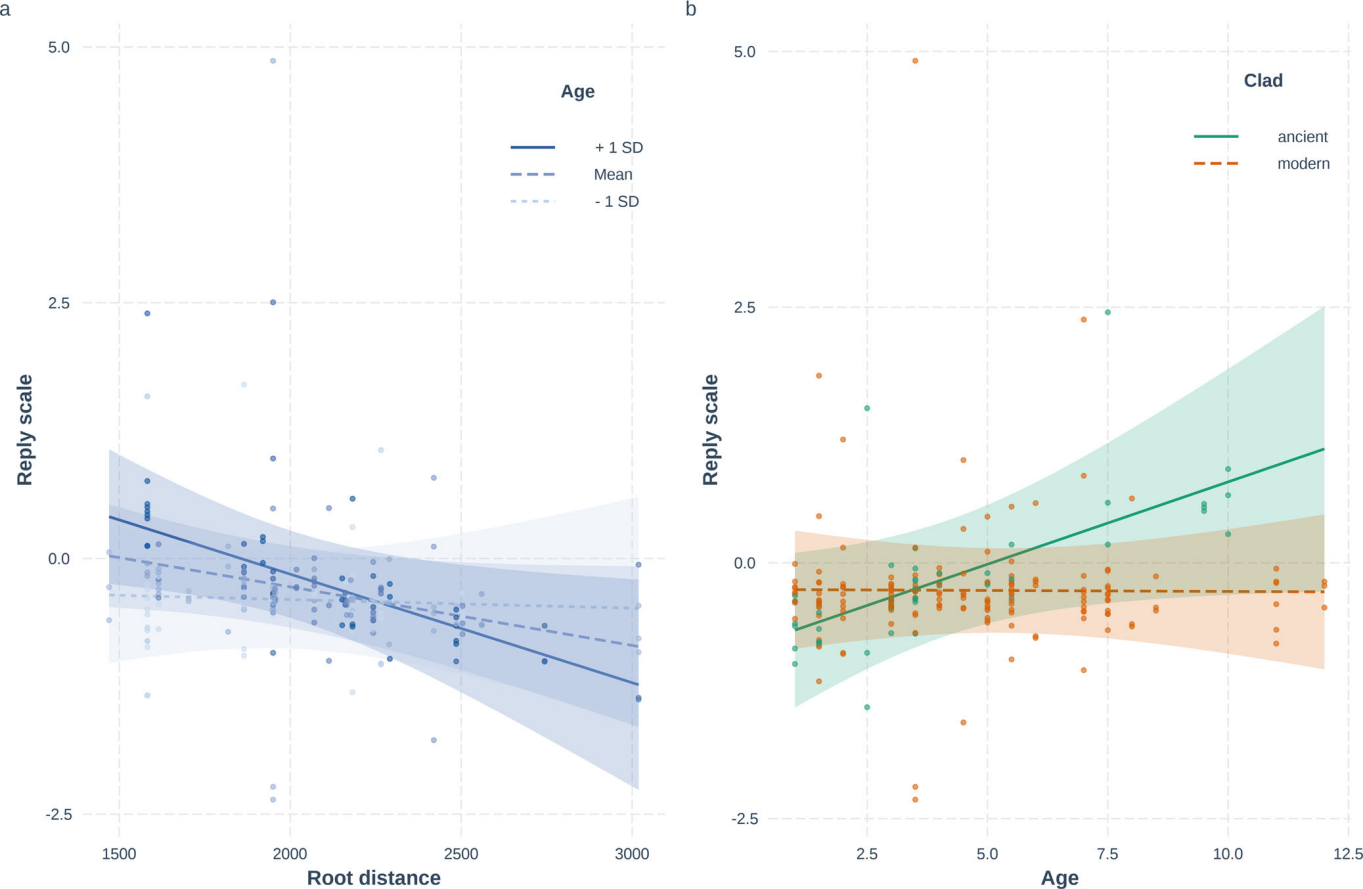
Effect of the interaction of Root distance/Breed group and age on the Reply scores (howling moaning, yelping, and bark-howling response). In **a**, *X*-axis shows the values of breeds’ genetic distance from wolves; the *Y*-axis shows the scores of the PCA factors, and the dots represent partial residuals, controlling for the effects of other variables in the model besides the plotted ones. The different types of lines represent the age categories: −1SD = 1.89 years, mean = 4.56 years, +1 SD = 7.23 years of age. In **b**, *X*-axis shows the age as a scaled variable, the *Y*-axis shows the scores of the PCA factors, and the dots represent the partial residuals from the model. The different types of lines represent the breed categories (ancient, modern).

Based on the PCA, howling, bark-howling, yelping, and moaning were all considered Reply vocalizations (Table 1). Root distance was associated with the dogs’ reply response in interaction with age (GLMM, LRT: *χ*^2^(1) = 8.565; *p* = 0.003, Table S1). We found a negative association with root distance, as more modern breeds had lower scores on the Reply scale, meaning they generally replied less often. Still, this effect was driven mainly by older individuals, and the relation was not significantly below the age of 5.2 years (Simple slopes analysis: at age 1.89 (−1SD): *β* ± SE = −0.01 ± 0.02; *t* = −0.54; *p* = 0.59, at mean age 4.56: *β* ± SE = 0.02 ± 0.01; *t* = 1.49; *p* = 0.14, at age 7.23 (+1SD): *β* ± SE = 0.05 ± 0.02; *t* = 2.58; *p* = 0.01; Fig. 1a). This relation is also present if we compare breeds grouped to ancient and modern ones (GLMM, LRT: *χ*^2^(1) = 10.828; *p* = 0.001; modern: *β* ± SE = 0.01 ± 0.02; *t* = 0.60; *p* = 0.55; ancient: *β* ± SE = −0.08 ± 0.03; *t* = −2.76; *p* = 0.01; Fig. 1b) showing that age had a significant positive effect in ancient breeds only on reply behaviour.

We also found an interaction effect of sex and reproductive status (GLMM, LRT: *χ*^2^(1) = 5.335; *p* = 0.021, Table S1) on the reply scale: neutered males replied longer than the intact ones; however, this difference was absent in females (Fig. S1, Table S2). We found no effect of the playback part on the replying.

#### Frequency of barking and whining

Two vocalisations, barking and whining, were excluded from the PCA scales due to crossloadings (for details, see the “Methods” section); thus, the effects of the investigated factors were tested on their frequency separately. We found that barking frequency is affected by the interaction of root distance and playback part (Poisson GzLMM, LRT: *χ*^2^(2) = 13.185; *p* = 0.001, Table S3). Although, according to the post-hoc test, the root distance effect was not significant in either part (Simple slopes analysis: Solo1: *β* ± SE = 0.59 ± 0.61; *z* = 0.97; *p* = 0.33, Chorus: *β* ± SE = 0.44 ± 0.59; *z* = 0.74; *p* = 0.46, Solo2: *β* ± SE = −0.34 ± 0.61; *z* = −0.56; *p* = 0.57), the trend in the second solo part differed from both the chorus (Tukey post-hoc test: *β* ± SE = 0.78 ± 0.27; *z* = 2.90; *p* = 0.01) and the first solo (Tukey post-hoc test: *β* ± SE = 0.93 ± 0.30; *z* = 3.01; *p* = 0.006): modern breeds bark more in Solo1 and Chorus, while in Solo2 their reactions decrease below the reactions of ancient breeds (Fig. S2a).

Concerning whining, we also found an interaction effect of root distance and playback part (Poisson GzLMM, LRT: *χ*^2^(2) = 7.331; *p* = 0.026), and again although the trends are not significant within the different parts (Simple slopes analysis: Solo1: *β* ± SE = −0.47 ± 0.39; *z* = −1.21; *p* = 0.23, Chorus: *β* ± SE = −0.15 ± 0.38; *z* = −0.38; *p* = 0.70, Solo2: *β* ± SE = −0.04 ± 0.38; *z* = −0.10; *p* = 0.92), the negative effect of root distance was stronger in the first solo than in the second (Tukey post-hoc test: *β* ± SE = −0.44 ± 0.17; *z* = −2.62; *p* = 0.02): in Solo1 modern breeds whine less than ancient ones (Fig. S2b).

### Frequency of stress behaviours

**Fig. 2:**
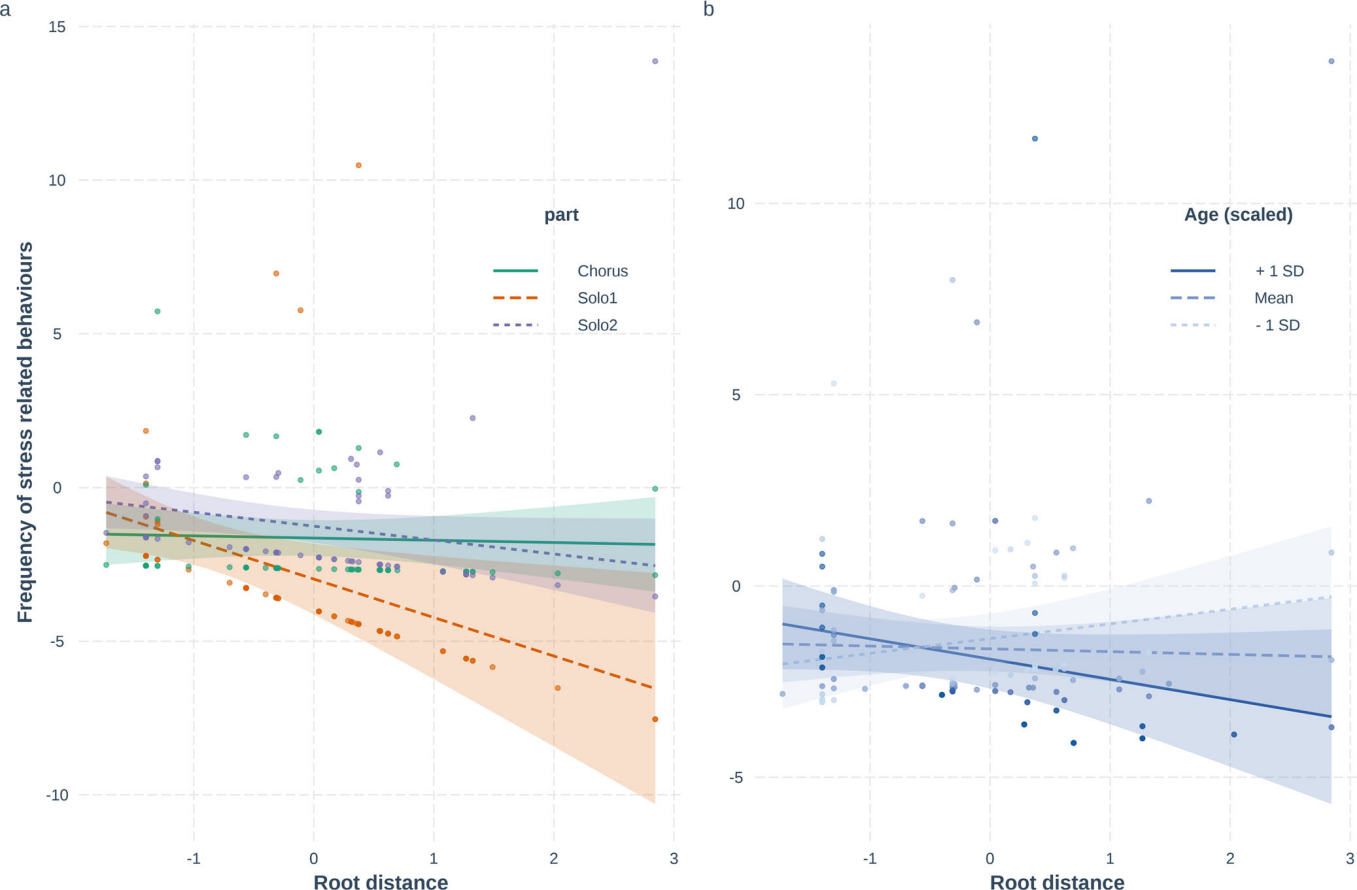
Effect of the interaction of Root distance and Playback part/Age on the frequency of stress behaviours. In **a**, *X*-axis shows the values of breeds’ genetic distance from wolves; the *Y*-axis shows the number of stress behaviours of the subjects, and the dots represent partial residuals. The different types of lines represent the playback parts (Solo1, Chorus, Solo2). In **b**, *X*-axis again shows the values of breeds’ genetic distance from wolves, the *Y*-axis shows the scores of the PCA factors, and the dots represent partial residuals, controlling for the effects of other variables in the model besides the plotted ones. The different types of lines represent the age categories: −1SD = 1.89 years, mean = 4.56 years, +1 SD = 7.23 years of age.

Stress behaviours (yawning, mouth licking, grooming, etc., see the complete list of behaviour variables in Supplementary Data 4) occurred in 32 out of 68 dogs (47%). From these 32 dogs 21 individuals vocalised (65,6%): 15,5% used agonistic vocalizations, 28,1% used owner-directed vocalizations, 37,5% used reply vocalizations, and 46,9 used stress vocalizations. We found a significant relationship between root distance and the frequency of stress behaviours in two ways. First, dogs from more ancient breeds showed more stress behaviours in both Solo parts than the modern breeds (Poisson GzLMM, LRT: *χ*^2^(2) = 6.345; *p* = 0.042, Solo1: *β* ± SE = −1.25 ± 0.50; *z* = −2.49; *p* = 0.01, Solo2: *β* ± SE = −0.45 ± 0.23; *z* = −1.93; *p* = 0.05). Second, in the Chorus playback, independently from the root distance, individuals showed as high frequency of stress behaviours as in the Solos (Chorus: *β* ± SE = −0.07 ± 0.25; *z* = −0.29; *p* = 0.77, Fig. 2b). Furthermore, we identified a negative relationship between root distance and stress behaviours in older dogs: dogs older than 8.3 years from more modern breeds showed significantly fewer stress behaviours than ancient breeds, while in young dogs, the occurrence of stress behaviours was similar in all breeds (Poisson GzLMM, LRT: *χ*^2^(1) = 5.113; *p* = 0.024, Simple slopes analysis: at age 1.89 (−1SD): *β* ± SE = 0.38 ± 0.30; *z* = 1.29; *p* = 0.20, at age 4.56 (mean): *β* ± SE = −0.07 ± 0.25; *z* = −0.29; *p* = 0.77, at age 7.23 (+1SD): *β* ± SE = −0.53 ± 0.34; *z* = −1.57; *p* = 0.12).

Results of the Attention scale are provided as Supplementary results and tables (Tables S5 and S6).

The main results are summarised in Table 3.

**Table 3:** Summary of the main results.

Behaviour variables	Effect of root distance in:		Playback part
	Younger dogs	Older dogs	
Reply scale	No difference under age 5.2 years	Ancient > Modern	No difference
Barking (frequency)	No difference	No difference	Root distance effect in: Solo1: Ancient < Modern Chorus: Ancient < Modern Solo2: No difference
Whining (frequency)	No difference	No difference	Root distance effect in: Solo1: Ancient > Modern
Stress behaviours (frequency)	No difference under age 8.3 years	Ancient > Modern	Root distance effect in: Solo1: Ancient > Modern Chorus: No difference Solo2: Ancient > Modern

## Discussion

Our results show that the genetic distance of domestic dog breeds from their last common ancestor with wolves (root distance), along with age, are robust predictors of their behavioural response to wolf howl playbacks causing differences in vocal reactions and stress behaviours. Specifically, older individuals of more ancient breeds’ reactions were characterised by howling and moaning, while young individuals, regardless of their genetic distance from the common ancestor, showed moderate reactions. Moreover, dogs responding with howling to the playbacks often showed stress behaviours.

Due to their genetic relationship with wolves, dogs’ reactions towards howling can be homologous to their closest living relatives. However, as the correlations with genetic distance from the common ancestor in the older dogs suggested, domestication impacted dogs’ vocal behaviour. Furthermore, dogs have a quite different social system than wolves^66^, live in a different environment with different selection forces, and our subjects were tested at an unfamiliar place, all of which could also affect their responsiveness. Hence, we will explore several different possible explanations for our results.

We hypothesised that answering with howling would negatively correlate with root distance, thus more modern breeds can be expected to howl less. We found this pattern, but only when considering the individual’s age. It seems that although howling is present in the repertoire of most breeds^48,50^—we can see this among our younger subjects too, which can be because of lack of selection against this vocalisation type—but it lost its functionality due to the loosened selection pressure caused by the changed social and asocial environment in modern breeds so they do not use it in adequate situations at an older age when it would be expected based on wolf studies. Results on dingoes (*Canis dingo*) seem to be in line with our genetic results and the hypothesis detailed above: they diverged from dogs as long as 8000 years ago^67^ and have a social structure more similar to wild canids, use howls extensively, e.g., to localise each other just like wolves^8,68^.

The genetic effect we found related to howling, that ancient breeds, namely at least the older individuals, howl more than modern breeds, seems to have an opposite pattern in the case of barking, what was suggested by our study and by previous ones too: while barking is used in only limited contexts by some ancient breeds (Siberian husky^36^, Basenji^69^, Chow-chow, Shar-pei^33^) like in dingoes^68^ and wolves too, it is widespread in modern dog breeds^33^. In line with this, we also found in our study that modern breeds bark more than ancient breeds in response to wolf howl playbacks, although this relationship appeared only in the first two playback parts out of the three. This barking result is not robust enough to draw unequivocal conclusions from it, but it is in line with the previously mentioned studies’ findings on barking usage in ancient and modern breeds^33,36,69^, and it may indicate that while ancient breeds rather answer with howls to the wolf howls, modern breeds may vocalise towards owners. This hypothesis requires further investigation. Furthermore, while barking can appear as a stereotypical behaviour in dogs, that is elicited by various, sometimes irrelevant stimuli or even without stimulus^70–72^, we know nothing about dog howling in this sense either. Based on our results, we do not suspect the howling in this test situation to be a stereotypic response. Although we did not test our subjects with other sound stimulus but the howling, in case of a stereotypical behaviour one would hypothesise a more enhanced howling reaction to the more intense and complex sound, the chorus, however, our results do not show this pattern.

An alternative possible hypothesis for more howling reactions in ancient breeds could be related to their original function, the way of working (group vs. solitary working), or their cooperation with humans during work the breeds were selected for, which all can affect their vocal behaviour, too. Ancient breeds were selected for pulling sleds (Siberian huskies, Alaskan malamutes, Chow-chow) and hunting (Salukis, Afghan hounds, Shiba Inu, Akita Inu, Basenji, Shar-pei Chow-chow), but some breeds were also used for guarding (Shar-pei, Akita Inu) and herding purposes (Chow chow, Shar-pei), too (The American Kennel Club, 2006). They primarily work in groups and in loose contact with humans, which can be related to ancient breeds using howling instead of barking. This pattern is the opposite in modern breeds, where barking is a universal vocalisation type, appearing in various contexts and inner states, and the breed functions are much more diverse. Some require more contact with humans, like herding dogs, retrievers, and gundogs. To confirm or reject this hypothesis, further investigation of the reactions of breed groups selected for different purposes (guarding, hunting, pulling sleds) or different working styles (solitary vs. group workers) to wolf howls would be needed. However, the collection of howler dogs seems a significant challenge in the case of some breed groups we also had to face during our data collection, which can be claimed as a limitation of our study regarding the number of subjects.

In addition, our results suggest that ancient breeds show a significantly higher frequency of stress behaviours than the more modern breeds and, accordingly, used more whines. This result on the stress behaviours may also confirm our hypothesis that more ancient breeds, due to their genetic relatedness, can process the information encoded in wolf howls better. A recent study with wolves showed that the contexts eliciting howling and anxious behaviour are highly overlapping. They can co-occur e.g. in presence of strange wolves, and the authors suggest that howling may have a resolving function in such situations^73^. Ancient breeds of our study might become stressed by intruding on a pack’s territory and use howling to sign their presence for the sake of avoidance. Another possible explanation for the elevated stress response in ancient breeds could be the found trend of a higher level of stress behaviours in ancient breeds during the warming up phase. There can be a higher sensitivity of these breeds to novel situations and to noises, which could elicit a higher level of stress even in the warming-up phase, and then it turned significant during the wolf howl playbacks, but until now, we haven’t found any studies confirming this hypothesis.

The genetic effects related to howling and stress behaviours seem to be significant only in older individuals: older dogs in ancient breeds were more responsive and replied with howling more than modern breeds and showed more stress signals, while this genetic effect was not significant in young individuals. Harrington and Mech found that the responsiveness of wolves can increase with social status, which in wild packs also correlates with age^3^. In our sample, the individuals from ancient breeds older than approximately 5.2 years had a higher tendency to reply with howls to the stimuli, hence making it unlikely that this age effect is linked to sexual/hormonal maturation but rather an experience- or some age-related personality effect might be a more plausible explanation^56,61,74,75^. Wolves may also become more aggressive and active in territory defence with ageing^54,55^. Furthermore, it is possible that this effect is a remnant of the wolves’ social behaviour and pack dynamics, as young adults tend to leave their family pack and form a new one or join another pack^8^, suggesting that individuals in this age range might have lower concerns upon hearing howls from unknown individuals. However, it is also possible—in line with the hypothesis described in the previous paragraph, that howling appearing with a higher level of stress is a fear reaction—that older dogs’ are more fearful, which was already suggested by previous studies^56,58^, but these speculations again require further testing.

Besides age, sex and reproductive status also affected the dogs’ reactions: neutered males replied with howling longer than intact males, while there was no difference between the two female groups. This result contradicts our hypothesis that males, especially intact males, will be more responsive. The howling reaction may be affected by the androgen hormones: In their study, Kaufmann et al.^76^ suggested that fear-related aggression can be influenced by the interplay of testosterone and cortisol: fear reactions are controlled by cortisol but can be inhibited by testosterone. In line with this, howling can be a sign of higher-level fear in castrated males, which can be, on the one hand, concordant with the result about the responsiveness and more stressed behaviour in genetically more related breeds, and on the other hand, in line with previous studies, suggesting that neutered males can be more fearful than intact ones^61,76^. An alternative possibility is that the effect of testosterone on the behaviour is suppressed by cortisol (called the dual-hormone hypothesis), which could have happened in this test situation, and therefore intact males did respond less vocally to the stimulus. Studies suggest the inhibitor effect of cortisol on the way between testosterone leads to the dominant behaviour across the downregulation of androgen receptors^77^. These theories and further assumptions (e.g., the interaction effect of breed and sexual status, the effect of personality like assertiveness or boldness, the impact of experiences like level of socialisation with other dogs) should be tested in the future to get closer to the understanding of howling behaviour in dogs.

Based on our results on vocal behaviour, we hypothesise that the common ancestor’s most prominent long-distance signal lost its communicative function in the human social niche, and probably a repertoire of exaggerated and diversified barks took its place in the communicative system of dogs^32,33^. Extending the range of subjects could give us the possibility to test this hypothesis: for example, in feral or pariah dogs, which do not socialise with humans as extensively as family dogs, and dingoes, which diverged from domestic dogs at an even earlier time point, the development of responses could be explored. The vocal behaviour of these canines, living outside the human social niche must be under different selection pressures, may be affected by these environmental and social factors more than by their genetic relatedness with the last common ancestor with wolves. An intriguing avenue for future work would also be to examine the effect of specific acoustic features of howls, which correspond to the caller’s sex, reproductive status, subspecies, or context.

## Methods

### Subjects

We tested 68 dogs from 28 breeds in this study. 17 dogs belonged to ancient breeds (Shiba inu, Siberian husky, Alaskan malamute, Akita inu, Shih tzu, Pekingese). Forty-eight dogs originated from registered breeders, and 20 dogs came from different sources: non-registered breeders or breed rescue centres, which were also reasonably presumed to be purebred (Table S4; To make sure that our main findings are sound and rule out a possible confounding effect of the 20 non-pedigree dogs, we tested our main effects on reply behaviours on dogs only with pedigree. We found the same trends as in the case of the whole subject sample, see Supplementary results). Based on the owners’ self-report, all participating dogs were known to howl in different contexts (Before testing all owners were asked whether their dog howls, in which context does it do and they also had to fill in an online questionnaire about their dog’s general vocal behaviour). This criterion ensured that the possible lack of howling behaviour during the test is not related to the lack of ability to howl in general. None of our subjects was experienced with a live wolf howling. Genetic distance from the common ancestor with wolves (root-distance) of the breeds involved in our study was approximated from the phylogenetic tree provided by Parker et al. ^38^ as *nex* tree Supplementary Data 6, using the ‘ape’ package^78^ by calculating the distance between each leaf of the tree and the root of the tree (based on the golden jackal as outgroup using function vcv). Distance values of each leaf containing the same breed were averaged, and this breed average was included as a continuous variable in our current analysis. The distribution of the subjects through the root distance spectrum was even. As a more conservative approach, we used a clad-based categorisation from Parker et al. (2017)^38^ and also compared ancient (or basal) and modern breed groups.

The sample included adult dogs between 1 and 12 years of both sexes, with different reproductive statuses, with no hearing problem based on the owners’ report (age: 4.57, mean ± 2.66 SD, 34 males, 34 females, 37 neutered/spayed (22 females, 15 males), for details, see Table S4). Intact females were tested at least two weeks before or after their heat period.

To ensure that there is no accidental correlation between the dogs’ age and their root distance, and exclude the possibility that the found interaction effects are due to this collinearity, we ran a robust correlation analysis using Winsorized Pearson correlation test (ggscatterplot from ggstatsplot package) due to the skewness of both variables. We found no significant correlation (for details, see Fig. S3).

### Behavioural test set-up

**Fig. 3:**
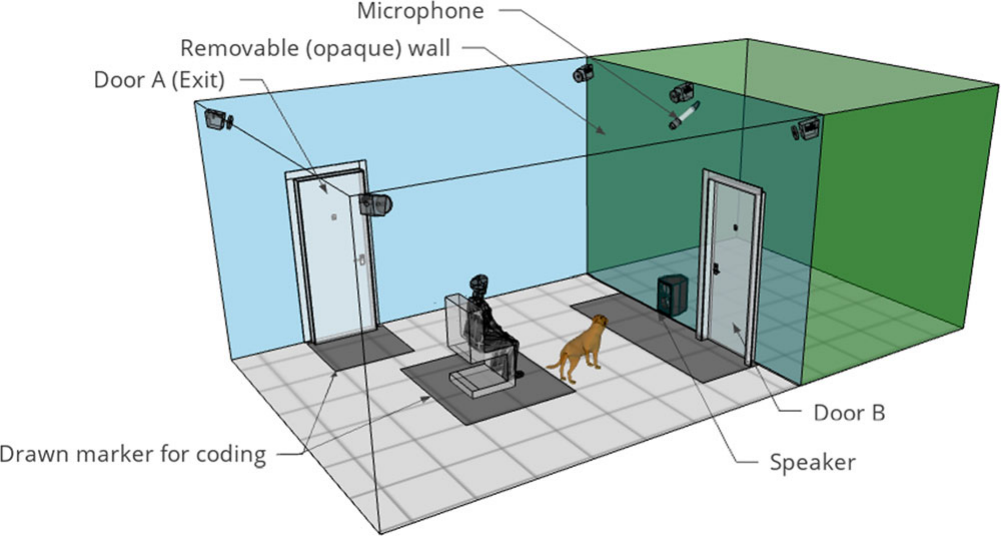
The setup of the testing room. The owner sat on a chair, listening to music  through headphones, and reading a book during the test. The dog moved freely in the room, and the stimulus was played from a speaker hidden behind the opaque, removable wall. Drawn markers served for coding the dogs’ proximity to the owner, sound source, and exit.

Dogs were tested at the Department of Ethology’s behaviour laboratory, a two-compartment room divided by a removable wooden half-wall containing a door. The dog and the owner stayed in the large compartment (6.27 m × 5.4 m), while a speaker (Technics SB-M300M2, 40–45,000 Hz, 60 W, 6 Ohm, 85 dB) was hidden behind the half-wall in the small part (5.2 m × 3 m, Fig. 3). Dogs’ behaviour (vocal-, motor- and postural-) was recorded by five IP cameras (Basler sca640–120gc) located in the large compartment beyond the ceiling (in the corners and one more in the middle of the removable wall) and one microphone (Sennheiser ME-62 with a K6 power module, located in the middle of the lab hanging from the ceiling, Fig. 3) connected to the PC through a Zoom H4n as a USB sound card. A chair was placed in the middle of the large compartment for the owner to sit on. The video and audio were recorded by a PC system located in a neighbouring room, and the playbacks were controlled from a separate PC using Adobe Audition 3.0.

### Procedure

The owner and their dog entered the testing room through *door A* with the experimenter. After giving a brief instruction, the experimenter left the room, which marked the starting point of the test. During the test, the dog moved freely in the room, the owner sat on a chair and listened to music through headphones while reading a book. The presence of the owner was needed to function as a secure base for the dog in this strange place, but he/she was asked to ignore the dog during the experiment to avoid giving potential behavioural cues which could alter the subject’s reaction to the stimulus. The first 1 min of the test was a familiarisation phase when dog could freely explore the lab. After this silent 1 min, the experimenter started to play the stimulus, which was a ~3-min-long sequence of wolf howls (mean volume was 50 dB with a peak at 60 dB measured from the owner’s chair). 30 s after the end of the stimulus, the experimenter stopped the video recording, and the test was finished. Please see examples in Supplementary Movies 1 and 2.

### Test sounds

**Fig. 4:**
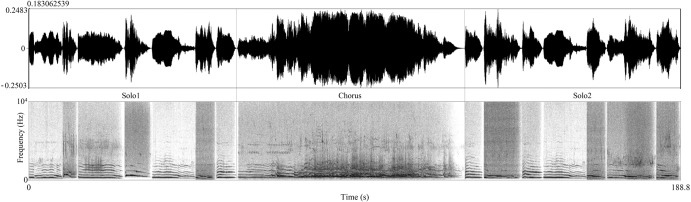
Construction of the howling stimulus. It consisted of three parts in a defined order: Solo1, Chorus and Solo2.

Considering that domestication might affect the acoustic structure and reduce the information content of dog howls, while breed selection might have further influence on the different breeds’ howls (see, e.g., baying behaviour in scent hounds), we decided to use wolf howls as stimuli to keep the potential information complexity in the playbacks similar for dogs from different breeds. Stimuli were built up from 3 parts: Two ~1-min-long series of solo wolf howls and a ~1-min-long wolf chorus howling between the two solo parts. There was no break or overlap between the three parts or between the individual solo howls, which number varied between 6 and 11 (Fig. 4). We aimed to test whether the simpler (solo) or rather the more complex form (chorus) elicit a response from the dogs. The purpose of the second solo part was to check whether the chorus causes sensitisation in the subjects and makes them respond more in contrast to the first solo part. We made 60 sessions from the collected 13 chorus howls and 80 solos, avoiding using the same solos more than once in the same session (please see an example of a howling session as Supplementary Audio File 1). Howls were drawn from male and female individuals of at least six subspecies of wolves (mixed and randomly chosen for the solo parts), and pack size ranged from 2 to 7 individuals in the case of chorus howls (Supplementary data 5). Each sample was converted to mono format, normalised to −23 dB (root-mean-square), and cleaned from most background noise (e.g., wind, low-frequency noise, except birdsong) using Adobe Audition with a high-pass FFT filter adjusted to the lowest frequency of the howl. The sounds that were too noisy or contained speech in the background were excluded from the sound pool.

### Video and data analysis

Videos were analysed in Solomon coder (András Péter^©^) with a 0.2 s time resolution. Coded behaviour variables are shown in Supplementary Data 4 with their definition and type. Behaviour variables were classified into groups, and while variables of different behaviour groups could appear simultaneously, variables of the same group could appear exclusively. Percentage data were used for statistical analysis, measured from the start to the end of the playback parts.

The frequency of other stress behaviours (yawning, mouth licking, stretching, grooming, shaking, urinating, defecating) during the 1-min-long playback intervals were counted as well. Furthermore, to assess the subject’s inner state before the howling stimulus, we analysed the frequency of stress signals in the warming-up phase (see Supplementary Results and Fig. S4). R studio (https://www.rstudio.com/) was used for the statistical analysis.

The coding reliability was determined using kappa statistics based on 20% of the sample recoded by an independent coder blind to the purpose of the tests. The average kappa value was 0.831 ± 0.066.

### Statistics and reproducibility

R studio (https://www.rstudio.com/) was used for the statistical analysis (R 4.0.0 using RStudio 1.2.5042). Time percentage was calculated for each behaviour separately (except in the case of Growl, which was merged with Growl-howl, and Whine, which was merged with Whine-howl), then they were combined into principal component scores by principal component analysis (PCA) (psych package, principal function with oblimin rotation). Rare events with low overall variance (Touch speaker, Other vocalisation) and variables with low sampling adequacy based on KMO analysis (Sit) or either low or cross-loadings were excluded iteratively (Explore speaker, Whine and Whine howl, Bark, Proximity to the sound). The number of components was determined in each iteration using parallel analysis (paran package). Internal consistency of the components was tested with standardised Cronbach alpha tests (alpha function, psych package). All alphas were above 0.5 and thus considered acceptable.

The components’ scores were transformed with Box-Cox transformation (‘boxcox’ function, ‘MASS’ package) if they differed from a normal distribution. General linear mixed models (GLMMs, ‘nlme’ package, ‘lme’ function, controlling for heteroscedasticity between playback parts) were used for analysing the principal components as separate response variables. In our main analysis, we tested effects on scores involving vocal behaviour (Reply and Agonistic calls) and Attention. The frequencies of stress signals were summed within each playback part and were analysed with a Generalised Linear Mixed Model with Poisson distribution and log-link, just as Barking and Whining that fell out from the PCA. All models included subject ID as a random factor, fixed effects of age (scaled), root distance (scaled)/breed group (ancient/modern), sex, reproductive status, playback part (Solo1, Chorus, Solo2) and also their interaction with root distance, and the interaction between sex and reproductive status. To avoid overfitting, we used backward elimination model selection based on Akaike information criterion (‘AIC’) (‘lmerTest’ package, drop1 function) eliminating the first interaction then main effects that did not contribute to the model fit significantly to find the final, parsimonious models (reported in the results) explaining the most variance in our data the simplest way. For post-hoc pairwise comparisons between factor levels and factor interactions, we used the Tukey test (‘emmeans’ package), while for analysing covariate interactions, Johnson–Neyman Interval tests (‘interact’ package) were applied. This so-called simple slope analysis tests how the trend of one focus covariate changes along the change of a modifying (interacting) covariate. It reports the breakpoints in the modifying variable where the trend of the focus variable becomes or loses significance, and the slope and statistical details at certain points (mean and ±1 SD of the modifying covariate).

### Reporting summary

Further information on research design is available in the Nature Portfolio Reporting Summary linked to this article.

## Supplementary information

Supplementary Information

Description of Additional Supplementary Files

Supplementary Data 1

Supplementary Data 2

Supplementary Data 3

Supplementary Data 4

Supplementary Data 5

Supplementary Data 6

Supplementary Audio 1

Supplementary Movie 1

Supplementary Movie 2

Reporting Summary

## Data Availability

All data generated or analysed during this study are included in this published article and its supplementary information files (Supplementary data 1–3).

## Code availability

Script used for the statistical analysis (R statistical environment (4.0.0) using RStudio (1.2.5042).) is provided as Supplementary data 6.
