# Supplementary material for: Genetic distance from wolves affects family dogs’ reactions towards howls
Source: Commun Biol. 2023 Feb 6;6:129. doi: 10.1038/s42003-023-04450-9 (PMC9902479; doi:10.1038/s42003-023-04450-9)
Supplement: Supplementary file 1 — Supplementary Information [file 42003_2023_4450_MOESM1_ESM.pdf]

SUPPLEMENT

Supplementary results

Age and root distance collinearity

The robust correlation analysis showed no significant correlation between dogs' age and their breed's genetic distance from the common ancestor with wolves (Figure S3). This strengthens our finding that individual's age modifies the root distance effects.

Stress in the habituation phase

We found only a non-significant negative trend effect (LRT: $\chi^2(2)=3.108$; $p=0.08$; Poisson GzLM: $\beta\pm SE=-0.343\pm0.201$; $z=-1.712$; $p=0.087$) suggesting that ancient breeds might have been slightly more stressed before the test due to the novel situation already (Figure S4).

	Stress indicators in habituation phase			
Predictors	Incidence Rate Ratios	CI	Statistic	p
(Intercept)	0.418	0.278 – 0.595	-4.526	<0.001
rd sc	0.709	0.472 – 1.038	-1.712	0.087
Observations	68			
R^2 Nagelkerke	0.066			
log-Likelihood	-60.738			

Other reactions

The Attention scale, which includes orientation to the sound, listening, and head tilting, decreased with the playback parts (LRT: $\chi^2(2)=66.575$; $p<0.001$, Table S5), and there was an interaction trend if we compare ancient and modern breeds: ancient breeds seemed to habituate slower than modern breeds (LRT: $\chi^2(2)= 4.998$; $p=0.082$, Table S6).

Pedigree vs non-pedigree dogs

Replying was affected by the interaction trend of root distance and age (LRT: $\chi^2(1)=3.231$; $p=0.072$; at age 1.89 (-1SD): $\beta\pm SE = -0.02\pm0.02$; $t=-0.98$; $p=0.33$, at mean age 4.56: $\beta\pm SE = 0.01\pm0.02$; $t=0.57$; $p=0.57$, at age 7.23 (+1SD): $\beta\pm SE = 0.04\pm0.03$; $t=1.59$; $p=0.12$).

Supplementary Tables & Figures

Table S1. Model details for the Reply scale

	Reply			
Predictors	Estimates	CI	Statistic	p
(Intercept)	1.095	1.049 – 1.142	45.548	<0.001
rd sc	0.021	-0.004 – 0.046	1.618	0.111
age sc	-0.003	-0.028 – 0.023	-0.227	0.821
sex [female]	-0.066	-0.143 – 0.012	-1.670	0.100
neuter [spayed/neutered]	-0.083	-0.153 – -0.012	-2.311	0.024
rd sc * age sc	0.042	0.015 – 0.069	3.050	0.003
sex [female] * neuter [spayed/neutered]	0.121	0.018 – 0.223	2.318	0.024
**Random Effects**				
σ^2	0.01			
$\tau_{00\ dog}$	0.01			
ICC	0.54			
N_{dog}	68			
Observations	204			
Marginal R^2 / Conditional R^2	0.179 / 0.620			
log-Likelihood	128.064			

Table S2 Post-hock pairwise comparison of replying among dogs of different sex and reproductive status

contrast	estimate	SE	df	t.ratio	p.value
Intact male-Neutered male	0.0828	0.0358	61	2.311	0.0242
Intact female-Spayed female	-0.0380	0.0379	61	-1.005	0.3189

Table S3. Model details for the frequency of barks

	Reply			
Predictors	Incidence Rate Ratios	CI	Statistic	p
(Intercept)	0.005	0.000 – 0.073	-3.872	<0.001
part [Solo1]	0.446	0.285 – 0.698	-3.537	<0.001
part [Solo2]	0.675	0.458 – 0.995	-1.983	0.047
rd sc	1.551	0.486 – 4.951	0.741	0.459
part [Solo1] * rd sc	1.159	0.761 – 1.766	0.689	0.491
part [Solo2] * rd sc	0.458	0.270 – 0.777	-2.896	0.004
**Random Effects**				
σ^2	5.63			
$\tau_{00\ dog}$	23.12			
ICC	0.80			
N_{dog}	67			
Observations	201			
Marginal R^2 / Conditional R^2	0.011 / 0.806			
log-Likelihood	-141.125			

Table S4. Subjects of the playback test with their individual features: sex, reproductive status, age (years); breed, pedigree, root distance score (genetic distance from the common ancestor with wolves), and breed group (ancient vs modern, based on Parker 2017).

ID	Sex	Reproductive status	Age (years)	Breed	Pedigree	Root distance	Clad
1	Male	Neutered	3	Shiba Inu	1	1470.25	Ancient
2	Male	Intact	9.5	Siberian husky	1	1582.1	Ancient
3	Male	Intact	4	Siberian husky	1	1582.1	Ancient
4	Female	Neutered	10	Siberian husky	1	1582.1	Ancient
5	Female	Neutered	1.5	Siberian husky	0	1582.1	Ancient
6	Female	Neutered	2.5	Siberian husky	0	1582.1	Ancient
7	Female	Intact	1	Siberian husky	1	1582.1	Ancient
8	Male	Neutered	7.5	Siberian husky	0	1582.1	Ancient
9	Male	Neutered	2	Siberian husky	1	1582.1	Ancient
10	Male	Neutered	5.5	Alaskan malamute	0	1614.9	Ancient
11	Male	Intact	1	Alaskan malamute	1	1614.9	Ancient
12	Female	Intact	3.5	Alaskan malamute	1	1614.9	Ancient

13	Male	Intact	3.5	Alaskan malamute	1	1614.9	Ancient
14	Female	Neutered	3	Akita Inu	1	1702.8	Ancient
15	Male	Neutered	3.5	Pekingese	0	1818.3	Ancient
16	Male	Intact	7.5	Airedale terrier	1	1864.667	Modern
17	Male	Intact	3.5	Airedale terrier	1	1864.667	Modern
18	Male	Intact	4	Airedale terrier	1	1864.667	Modern
19	Male	Intact	2	Airedale terrier	1	1864.667	Modern
20	Female	Intact	1.5	Airedale terrier	1	1864.667	Modern
21	Female	Intact	5.5	Airedale terrier	1	1864.667	Modern
22	Male	Intact	12	Briard	1	1920.0	Modern
23	Female	Neutered	3	Dachshund	0	1949.8	Modern
24	Male	Neutered	7.5	Dachshund	0	1949.8	Modern
25	Male	Intact	5	Dachshund	0	1949.8	Modern
26	Male	Neutered	3.5	Dachshund	0	1949.8	Modern
27	Female	Intact	1	Dachshund	0	1949.8	Modern
28	Female	Neutered	7	Dachshund	1	1949.8	Modern
29	Female	Neutered	7	Dachshund	1	1949.8	Modern
30	Female	Intact	3	Dachshund	1	1949.8	Modern
31	Female	Neutered	3.5	Shih Tzu	0	1955.6	Ancient
32	Male	Neutered	5	Shih Tzu	0	1955.6	Ancient
33	Male	Intact	5.5	Belgian shepherd	1	2018.333	Modern
34	Male	Intact	5.5	Great Pyrenees	1	2070.2	Modern
35	Male	Intact	1	Great Pyrenees	1	2070.2	Modern
36	Male	Intact	4	Great Pyrenees	1	2070.2	Modern
37	Male	Intact	5.5	Great Pyrenees	1	2070.2	Modern
38	Female	Intact	5.5	Borzoi	1	2113.3	Modern
39	Female	Intact	2	Borzoi	1	2113.3	Modern
40	Male	Neutered	11	Jack Russell	0	2152.1	Modern
41	Female	Intact	1.5	Great Dane	1	2160.8	Modern
42	Female	Neutered	7	Great Dane	0	2160.8	Modern
43	Female	Neutered	4.5	Yorkshire terrier	1	2165.4	Modern
44	Female	Intact	4.5	Weimaraner	1	2177.0	Modern
45	Male	Intact	8	Labrador retriever	0	2182.3	Modern
46	Male	Neutered	1.5	Labrador retriever	0	2182.3	Modern
47	Female	Neutered	3	Labrador retriever	0	2182.3	Modern
48	Female	Neutered	1	Labrador retriever	1	2182.3	Modern
49	Female	Neutered	2	Rottweiler	0	2240.7	Modern
50	Female	Neutered	5	Hungarian vizsla	1	2242.857	Modern
51	Female	Neutered	5	Hungarian vizsla	1	2242.857	Modern
52	Male	Intact	7.5	Hungarian vizsla	1	2242.857	Modern
53	Female	Intact	1.5	German shepherd	1	2265.4	Modern

54	Male	Neutered	2	German shepherd	1	2265.4	Modern
55	Male	Intact	6	German shepherd	1	2265.4	Modern
56	Male	Neutered	3.5	Miniature poodle	1	2289.7	Modern
57	Female	Neutered	11	West highland	1	2291.4	Modern
58	Female	Neutered	4.5	English cocker	1	2420.0	Modern
59	Male	Neutered	3.5	English cocker	1	2420.0	Modern
60	Female	Neutered	7.5	Beagle	1	2485.5	Modern
61	Female	Intact	8.5	Beagle	0	2485.5	Modern
62	Female	Neutered	3	Beagle	1	2485.5	Modern
63	Female	Neutered	1.5	Border collie	1	2504.0	Modern
64	Female	Neutered	5	Border collie	1	2504.0	Modern
65	Male	Intact	4	Shetland sheepdog	1	2560.1	Modern
66	Male	Neutered	7	Boxer	1	2744.1	Modern
67	Female	Neutered	6	Bull terrier	0	3018.9	Modern
68	Male	Neutered	3	Bull terrier	1	3018.9	Modern

Table S5. Post-hoc pairwise comparison of attention among playback parts

contrast	estimate	SE	df	t. ratio	p.value
Chorus - Solo1	-0.580	0.105	134	-5.529	<.0001
Chorus - Solo2	0.548	0.103	134	5.309	<.0001
Solo1 - Solo2	1.128	0.117	134	9.620	<.0001

Table S6. Post-hoc pairwise comparison of attention among playback parts and breed groups

	contrast	estimate	SE	df	t.ratio	p.value
ancient	Chorus - Solo1	-0.360	0.208	132	-1.731	modern
	Chorus - Solo2	0.928	0.201	132	4.611	<.0001
	Solo1 - Solo2	1.288	0.235	132	5.476	<.0001
modern	Chorus - Solo1	-0.653	0.120	132	-5.441	<.0001
	Chorus - Solo2	0.422	0.116	132	3.627	0.0012
	Solo1 - Solo2	1.075	0.136	132	7.912	<.0001

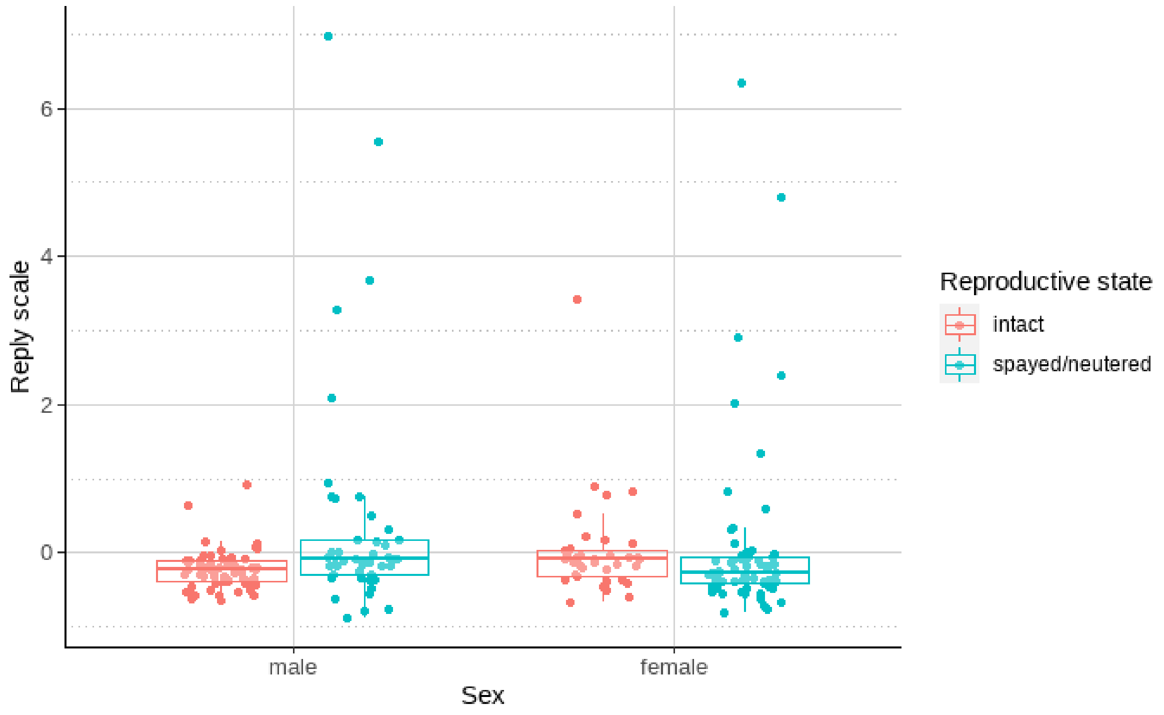

Figure S1. Effect of sex and reproductive status on the Replying scale. Each dot represents the data of an individual dog. Box plots show the medians (horizontal lines), upper and lower quartiles (boxes) and lowest and highest values within 1.5 times the interquartile range (whiskers). Colours indicate the reproductive status (intact, spayed/neutered) within the sex categories (males, females).

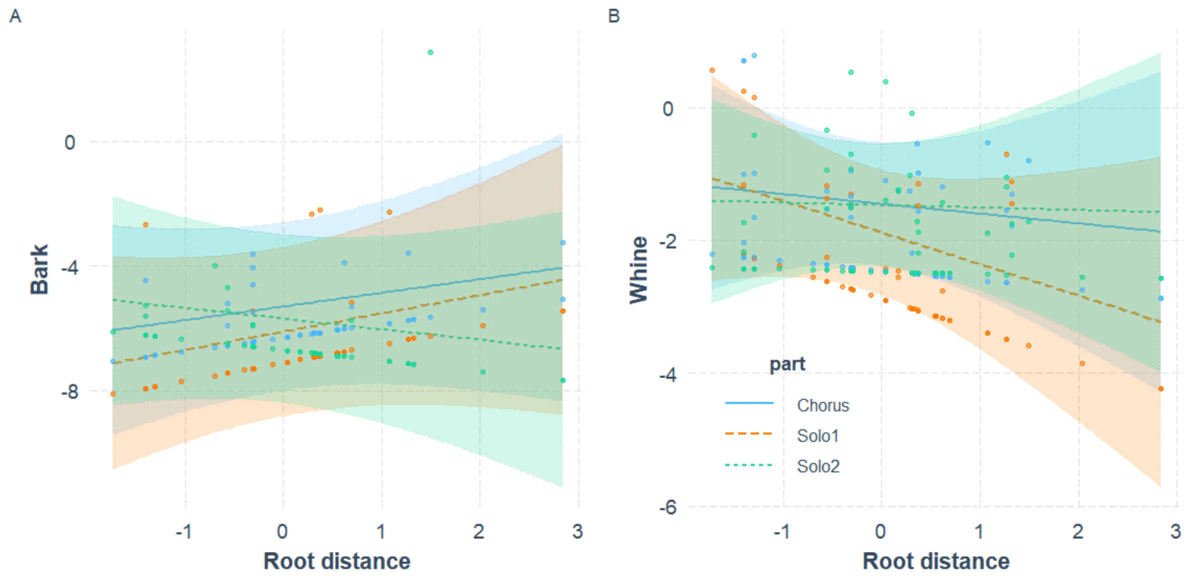

Figure S2. Effect of root distance and playback part on the frequency of Barking/Whining. X axis shows the root distance scores of the tested dogs' breeds, the y axis shows the frequency of the a, Barking, b, Whining, while the dots represent partial residuals. The different types of lines represent the three playback parts (Solo1, Chorus, Solo2).

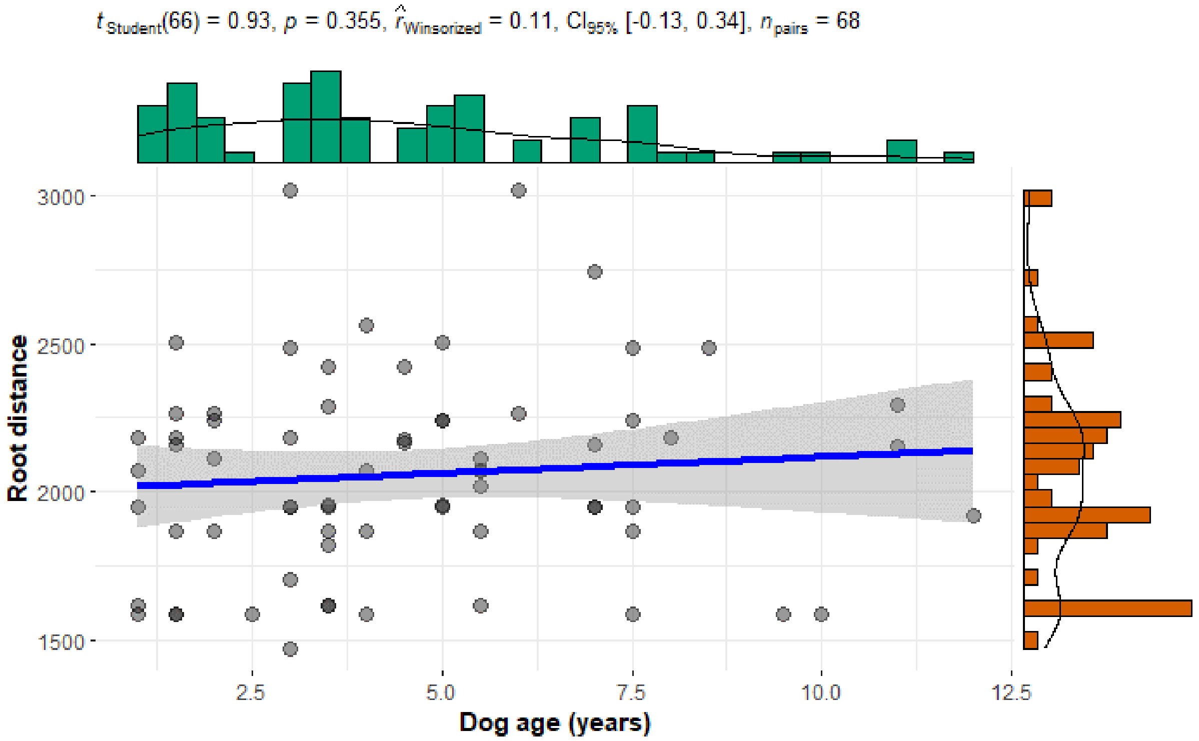

Figure S3. Result of collinearity test between age and root distance. The X axis shows the age of the tested dogs, the Y axis shows the root distance scores of the tested dogs' breeds. Statistics calculated and plot created by ggstatsplot package, ggscatterstats function

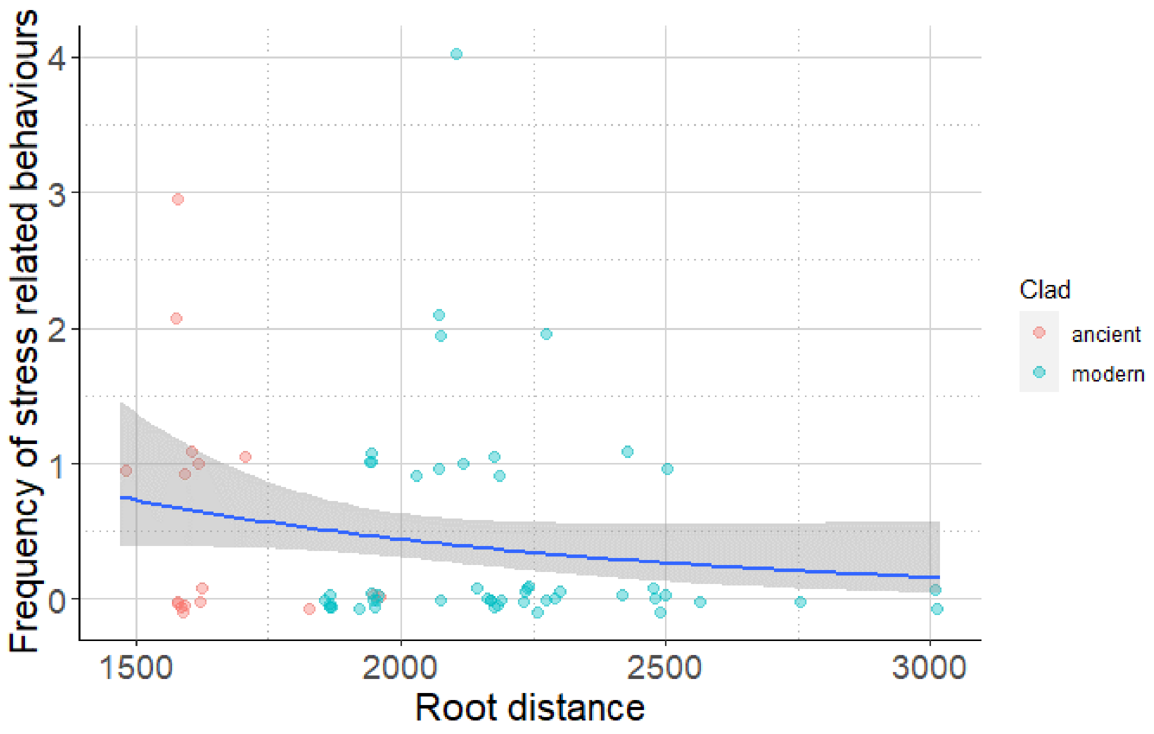

Figure S4. Frequency of stress reactions during the habituation phase in the tested breeds. The X axis shows the root distance scores of the tested dogs' breeds, the Y axis shows the frequency of stress behaviours. Each dot represents the data of an individual dog. Colours indicate the two breed groups (ancient, modern).
