# Supplementary material for: Genetic distance from wolves affects family dogs’ reactions towards howls
Source: Commun Biol. 2023 Feb 6;6:129. doi: 10.1038/s42003-023-04450-9 (PMC9902479; doi:10.1038/s42003-023-04450-9)
Supplement: Supplementary file 2 — Description of Additional Supplementary Files [file 42003_2023_4450_MOESM2_ESM.pdf]

Description of Additional Supplementary Files

File name: Supplementary Data 1
Description: Raw data used for figures and main statistical analysis.

File name: Supplementary Data 2
Description: Raw data used for figures and main statistical analysis.

File name: Supplementary Data 3
Description: Raw data used for figures and main statistical analysis.

File name: Supplementary Data 4
Description: Behaviour variable groups with variables and definition.

File name: Supplementary Data 5
Description: Information about the wolf howlings which were used for the playbacks.

File name: Supplementary Data 6
Description: R script.

File name: Supplementary Audio 1
Description: Sound stimulus sample.

File name: Supplementary Movie 1
Description: Test video (Ancient breed).

File name: Supplementary Movie 2
Description: Test video (Modern breed).
