# Supplementary material for: Genetic distance from wolves affects family dogs’ reactions towards howls
Source: Commun Biol. 2023 Feb 6;6:129. doi: 10.1038/s42003-023-04450-9 (PMC9902479; doi:10.1038/s42003-023-04450-9)
Supplement: Supplementary file 6 — Supplementary Data 4 [file 42003_2023_4450_MOESM6_ESM.docx]

**Supplementary Data 4.** Behaviour variable groups with variables and definition

| Variable Group | Variable | Definition | Type |
| --- | --- | --- | --- |
| Exploration | Exploring_lab | Dog looking around and/or sniffing in the lab, exploration | Time percentage(s) |
| Orientation | Orient to owner | Dog looking towards/at the owner | Time percentage(s) |
|  | Orient to sound | Dog looking towards the sound source | Time percentage(s) |
|  | Orient to exit | Dog looking to/towards the door A | Time percentage(s) |
| Others | Listening | From the start point to the end of the playback, the dog shows hearing behaviour: ear moving, listening | Time percentage(s) |
|  | Head tilting | dog is tilting its head | Time percentage(s) |
| Stress behaviours | Urinate | dog is peeing | Frequency |
|  | Defecate | dog is pooing | Frequency |
|  | Shaking | dog is shaking its body | Frequency |
|  | Mouth licking | dog is licking its mouth | Frequency |
|  | Stretching | dog is stretching its body | Frequency |
|  | Grooming | dog is licking, chewing itself | Frequency |
|  | Yawn | Dog is yawing. | Frequency |
|  | Prox to owner | Dog is close to the owner (Inside the drawn marker) | Time percentage(s) |
| Proximity | Prox to speaker | Dog is close to the speaker (Inside the drawn marker) | Time percentage(s) |
|  | Prox to exit | Dog is close to the door A (In what they entered with the owner, (Inside the drawn marker)) | Time percentage(s) |
| Moving | Stand | Dog is standing | Time percentage(s) |
|  | Move | Dog is moving: run, trot, walk, step, sit down, stand up, jump etc. | Time percentage(s) |
|  | Sit | Dog is sitting | Time percentage(s) |
|  | Lie | Dog is lying | Time percentage(s) |
| Vocalisation | Howl | The dog is howling (long tonal sound) | Time percentage(s) |
|  | Moan | Dog is moaning (sound type between growling and whining: moderately long, low pitch, with frequency modulations) | Time percentage(s) |
|  | Bark | Dog is barking (short tonal sound) | Time percentage(s) |
|  | Woof | Dog is woofing (Very short bark-like sound, but always noisy) | Time percentage(s) |
|  | Whine | Dog is whining (short or long high-pitched sound) | Time percentage(s) |
|  | Growl | Dog is growling (long noisy sound) | Time percentage(s) |
|  | Yelp | Dog is barking (short tonal high-pitched sound) | Time percentage(s) |
|  | Bark-howl | Barking, which transforms to howling without any break | Time percentage(s) |
|  | Whine-howl | Whining which transforms to howling without any break | Time percentage(s) |
|  | Growl-howl | Growling, which transforms to howling without any break | Time percentage(s) |
| Test markers | Start | Start point of the test, when the door closes behind the experimenter. | point |
|  | Stop | The end of the test. Half a minute after the last playback. | point |
|  | Solo_on | The start point of the solo playback (Based on the spectrogram and sound) | point |
|  | Solo_off | End of the solo playback (Based on the spectrogram and sound) | point |
|  | Chorus_on | The start point of the chorus playback (Based on the spectrogram and sound) | point |
|  | Chorus_off | End of the chorus playback (Based on the spectrogram and sound) | point |
