# Supplementary material for: Genetic distance from wolves affects family dogs’ reactions towards howls
Source: Commun Biol. 2023 Feb 6;6:129. doi: 10.1038/s42003-023-04450-9 (PMC9902479; doi:10.1038/s42003-023-04450-9)
Supplement: Supplementary file 7 — Supplementary Data 5 [file 42003_2023_4450_MOESM7_ESM.docx]

**Supplementary data 5.** Information about the wolf howlings which were used for the playbacks

| Type | Call id | Subspecies | Sex | Age (years) | Context |
| --- | --- | --- | --- | --- | --- |
| Solo | 14 | Eastern wolf | Unknown | Unknown | Unknown |
| Solo | 16 | Eastern wolf | Unknown | Unknown | Unknown |
| Solo | 18 | Eastern wolf | Unknown | Unknown | Unknown |
| Solo | 22 | Eastern wolf | Unknown | Unknown | Unknown |
| Solo | 23 | Eastern wolf | Unknown | Unknown | Unknown |
| Solo | 24 | Eastern wolf | Unknown | Unknown | Unknown |
| Solo | 25 | Eastern wolf | Unknown | Unknown | Unknown |
| Solo | 26 | Eastern wolf | Unknown | Unknown | Unknown |
| Solo | 27 | Eastern wolf | Unknown | Unknown | Unknown |
| Solo | 30 | Eastern wolf | Unknown | Unknown | Unknown |
| Solo | 32 | Eastern wolf | Unknown | Unknown | Unknown |
| Solo | 34 | Eastern wolf | Unknown | Unknown | Unknown |
| Solo | 74 | Eastern wolf | Male | 1 | Pack member temporally removed from Pack |
| Solo | 1 | European subspecies (Eurasian or Russian wolf) | Male | Unknown | Unknown |
| Solo | 2 | European subspecies (Eurasian or Russian wolf) | Male | Unknown | Unknown |
| Solo | 3 | European subspecies (Eurasian or Russian wolf) | Male | Unknown | Unknown |
| Solo | 4 | European subspecies (Eurasian or Russian wolf) | Male | Unknown | Unknown |
| Solo | 5 | European subspecies (Eurasian or Russian wolf) | Male | Unknown | Unknown |
| Solo | 6 | European subspecies (Eurasian or Russian wolf) | Male | Unknown | Unknown |
| Solo | 7 | European subspecies (Eurasian or Russian wolf) | Male | Unknown | Unknown |
| Solo | 8 | European subspecies (Eurasian or Russian wolf) | Male | Unknown | Unknown |
| Solo | 9 | European subspecies (Eurasian or Russian wolf) | Male | Unknown | Unknown |
| Solo | 10 | European subspecies (Eurasian or Russian wolf) | Male | Unknown | Unknown |
| Solo | 11 | European subspecies (Eurasian or Russian wolf) | Male | Unknown | Unknown |
| Solo | 12 | European subspecies (Eurasian or Russian wolf) | Male | Unknown | Unknown |
| Solo | 13 | European subspecies (Eurasian or Russian wolf) | Male | Unknown | Unknown |
| Solo | 15 | European subspecies (Eurasian or Russian wolf) | Male | Unknown | Unknown |
| Solo | 17 | European subspecies (Eurasian or Russian wolf) | Male | Unknown | Unknown |
| Solo | 19 | European subspecies (Eurasian or Russian wolf) | Male | Unknown | Unknown |
| Solo | 20 | European subspecies (Eurasian or Russian wolf) | Male | Unknown | Unknown |
| Solo | 20 | European subspecies (Eurasian or Russian wolf) | Male | Unknown | Unknown |
| Solo | 31 | European subspecies (Eurasian or Russian wolf) | Male | Unknown | Unknown |
| Solo | 35 | European subspecies (Eurasian or Russian wolf) | Male | Unknown | Unknown |
| Solo | 36 | European subspecies (Eurasian or Russian wolf) | Male | Unknown | Unknown |
| Solo | 37 | European subspecies (Eurasian or Russian wolf) | Male | Unknown | Unknown |
| Solo | 38 | European subspecies (Eurasian or Russian wolf) | Male | Unknown | Unknown |
| Solo | 79 | North-western wolf | Male | 2 | Spontaneous howling in captivity |
| Solo | 45 | Eurasian wolf | Female | 10 | Spontaneous howling in captivity, before chorus, |
| Solo | 46 | Eurasian wolf | Female | 10 | Spontaneous howling in captivity, before chorus, |
| Solo | 47 | Eurasian wolf | Female | 10 | Spontaneous howling in captivity, before chorus, |
| Solo | 48 | Eurasian wolf | Female | 10 | Spontaneous howling in captivity, before chorus, |
| Solo | 49 | Eurasian wolf | Female | 10 | Spontaneous howling in captivity, before chorus, |
| Solo | 50 | Eurasian wolf | Female | 10 | Spontaneous howling in captivity, before chorus, |
| Solo | 51 | Eurasian wolf | Female | 10 | Spontaneous howling in captivity, before chorus, |
| Solo | 52 | Eurasian wolf | Female | 10 | Spontaneous howling in captivity, before chorus, |
| Solo | 53 | Eurasian wolf | Female | 10 | Spontaneous howling in captivity, before chorus, |
| Solo | 54 | Eurasian wolf | Female | 10 | Spontaneous howling in captivity, before chorus, |
| Solo | 55 | Eurasian wolf | Female | 10 | Spontaneous howling in captivity, before chorus, |
| Solo | 56 | Eurasian wolf | Female | 10 | Spontaneous howling in captivity, before chorus, |
| Solo | 57 | Eurasian wolf | Female | 10 | Spontaneous howling in captivity, before chorus, |
| Solo | 58 | Eurasian wolf | Female | 10 | Spontaneous howling in captivity, before chorus, |
| Solo | 59 | Eurasian wolf | Female | 10 | Spontaneous howling in captivity, before chorus, |
| Solo | 60 | Eurasian wolf | Female | 10 | Spontaneous howling in captivity, before chorus, |
| Solo | 61 | Eurasian wolf | Female | 10 | Spontaneous howling in captivity, before chorus, |
| Solo | 73 | North American subspecies (no more information) | Male | 4,5 | Spontaneous howling in captivity |
| Solo | 90 | North American subspecies (no more information) | Female | 4,5 | Pack member temporally removed from Pack |
| Solo | 62 | Tundra wolf | Female | 3,5 | Pack member temporally removed from Pack |
| Solo | 63 | Tundra wolf | Female | 3,5 | Pack member temporally removed from Pack |
| Solo | 65 | Tundra wolf | Male | 3,5 | Pack member temporally removed from Pack |
| Solo | 66 | Tundra wolf | Male | 3,5 | Pack member temporally removed from Pack |
| Solo | 67 | Tundra wolf | Male | 3,5 | Pack member temporally removed from Pack |
| Solo | 68 | Tundra wolf | Male | 3,5 | Pack member temporally removed from Pack |
| Solo | 69 | Tundra wolf | Male | 3,5 | Pack member temporally removed from Pack |
| Solo | 76 | Tundra wolf | Male | 3,5 | Spontaneous howling in captivity |
| Solo | 77 | Tundra wolf | Male | 3,5 | Spontaneous howling in captivity |
| Solo | 87 | Tundra wolf | Male | 3,5 | Pack member temporally removed from Pack |
| Solo | 70 | North-western wolf | Male | 1 | Spontaneous howling in captivity |
| Solo | 71 | North-western wolf | Female | 1 | Spontaneous howling in captivity |
| Solo | 72 | North-western wolf | Female | 1 | Spontaneous howling in captivity |
| Solo | 75 | North-western wolf | Female | 1 | Pack member temporally removed from Pack |
| Solo | 78 | North-western wolf | Male | 1 | Spontaneous howling in captivity |
| Solo | 80 | North-western wolf | Male | 1 | Spontaneous howling in captivity |
| Solo | 81 | North-western wolf | Male | 1 | Spontaneous howling in captivity |
| Solo | 82 | North-western wolf | Male | 1 | Pack member temporally removed from Pack |
| Solo | 83 | North-western wolf | Male | 1 | Pack member temporally removed from Pack |
| Solo | 84 | North-western wolf | Male | 1 | Pack member temporally removed from Pack |
| Solo | 85 | North-western wolf | Female | 1 | Pack member temporally removed from Pack |
| Solo | 86 | North-western wolf | Female | 1 | Pack member temporally removed from Pack |
| Solo | 88 | North-western wolf | Female | 1 | Pack member temporally removed from Pack |
| Solo | 89 | North-western wolf | Female | 1 | Pack member temporally removed from Pack |
| Chorus | 5 | Arctic, Great Plains, North-western wolves | 6 wolves | 9,9,5,5,1,1 | Pack rally after resting |
| Chorus | 4 | Eastern and North-western wolves | 3 M, 3 F | 2,2,1,1,1,1 | Elicited by a plane taking off |
| Chorus | 2 | European subspecies (no more information) | 3 M, 2 F | 4,4,4,1 | Spontaneous howling |
| Chorus | 3 | European subspecies (no more information) | 3 M, 2 F | 4,4,4,1 | Pack assembles |
| Chorus | 10 | Eurasian wolves | 1 M, 1 F | 11,11 | Elicited by human imitation |
| Chorus | 14 | Eurasian wolves | 7 wolves | All >= 1 year | Elicited by human imitation |
| Chorus | 15 | Eurasian wolves | 7 wolves | All >= 1 year | Elicited by human imitation |
| Chorus | 16 | Eurasian wolves | 3 M, 1 F | One yearling male and the other adults (>=2 ) | Elicited by human imitation |
| Chorus | 19 | North-western wolves | 1 M, 1 F | 10, 10 | Unknown |
| Chorus | 20 | North-western wolves | 1 M, 2 F | 5,5,5 | Unknown |
| Chorus | 25 | North-western wolves | Unknown | Unknown | Elicited by air raid warning siren |
| Chorus | 26 | North-western wolves | Unknown | Unknown | Elicited by air raid warning siren |
| Chorus | 18 | North-western wolves and Arctic wolf | 3 M, 2 F | 10,8,5,5,5 | Unknown |
